# Association between Dietary Patterns, Breakfast Skipping and Familial Obesity among a Sample of Egyptian Families

**DOI:** 10.3889/oamjms.2016.050

**Published:** 2016-04-21

**Authors:** Nayera E. Hassan, Salwa M. El Shebini, Nihad H. Ahmed

**Affiliations:** 1 *Biological Anthropology Department, National Research Centre, Cairo, Egypt*; 2 *Nutrition and Food Science Department, National Research Centre, Cairo, Egypt (Affiliation ID: 60014618)*

**Keywords:** Familial obesity, anthropometry, dietary habits, breakfast

## Abstract

**AIM:**

To examine the association between dietary patterns, behaviors and the prevalence of familial obesity.

**SUBJECTS AND METHODS:**

Eighty three families, shared as volunteers comprised of 83 mothers and 155 offspring. Anthropometric measurements were reported including height and weight. Body mass index (BMI), weight/height, and weight/height Z score were calculated. Pattern of food intake was obtained by means of dietary interview consisting of a 24 hour recall, and a food frequency questionnaire.

**RESULTS:**

Data revealed that obesity was high among mothers reached 91.6% while obesity in the offspring was 24.5%. According to prevalence of obesity, families were divided to 4 groups, 8.43% of families were of normal weight, and 20.48% were obese. Food frequency consumption rate and food analysis revealed unhealthy food intake, especially in obese families. All groups reported high rate intake of sweets, pastries and beverage. Calories, carbohydrate, cholesterol and sodium were higher than the RDA in all mother’s groups, and adolescent group (2) compared to low daily intake of micronutrients especially calcium and vitamin D in all groups. More than half of all mothers and offspring skipped breakfast.

**CONCLUSION:**

Results of this study suggest that familial obesity increases the risk of offspring being obese, dietary habits might be involved in the development of obesity.

## Introduction

Familial history of obesity and certain dietary habits are risk factors for obesity. Parental behaviors forming part of the home and family environmental sphere of influence within the Socio-Ecological Model [1]: include practices, such as making healthy foods available, establishing expectations for healthful food consumption, and setting a good example. These practices have been positively associated with overall diet quality of youth [2-5].

Thompson et al. (2010) [6] reported that meal skipping has not been examined as a potential risk factor among numerous behavioral changes lead tothe prevalence of overweight and obesity especially in youth living in nations undergoing rapid economic and social change.

Regular breakfast consumption can have a multitude of positive health benefits, yet young people are more likely to skip breakfast than any other meal. Given the evidence that dietary behaviors established in childhood and adolescence track into adulthood along with evidence that breakfast skipping increases with age, identifying correlates of children’s and adolescent’s breakfast behaviors is imperative [7].

The role of micronutrients in energy balance and obesity remains understudied. However, many researchers studied the effect of calcium on body weight. Epidemiological and cross-sectional investigations began to identify calcium intake as a dietary constituent that was inversely related to body weight and body fat levels [8-10]. These initial observations were followed by mechanistic studies in animal models. One hypothesis generated postulates that low calcium intake leads to increased intracellular calcium levels due to a change in circulating calcium-regulating hormones, particularly 1, 25-dihydroxyvitamin D and parathyroid hormone. High intracellular calcium levels, in turn, act to reduce lipolysis and increase lipogenesis in adiposities [11]. Increasing dietary calcium is thought to inhibit these effects and facilitate fat loss [12]. The results imply that high vitamin D and Ca intakes activate the Ca(2+) -mediated apoptotic pathway in adipose tissue. Targeting this pathway with vitamin D and Ca supplementation could contribute to the prevention and treatment of obesity [13].

The aim of the study was to examine the association between dietary patterns, behaviorsand the prevalence of familial obesity particularlyamong mothers and their offspring

## Subjects and Methods

Eighty three families, shared as volunteers in thiscohort prospective study. These families comprised of 83 mothers and 155 offspring, (82 children and 73 adolescents). They were enrolled in a program for nutritional education through a project funded by National Research Centre (NRC) Egypt, 2013-2016: titled “ Familiar Overweight and Obesity in Children and Adolescents: Diagnostic Clinical, Behavioral, Genetic and Biochemical Markers and Intervention”, after taking approval from Ethical Committee of NRC (Registration Number is 13- 168) and written informed consent from each of them.

### 

#### Anthropometric parameters

Relevant anthropometric measurements were reported including height and weight using standardized equipment, and following the recommendations of the International Biological Program [14]. Body mass index (BMI) was calculated (weight in kg/height in meter^2^). Weight/height, and weight/height z score were calculated for children. Then, families were classified to four groups according to distribution of Obesity among mothers and offspring (BMI for mother ≥ 30, for children and adolescents ≥ 95 percentile, z score for children ≥ +2 SD)

#### Dietary recalls

Information on each mother’s usual pattern of food intake was obtained; the same for the adolescents, while data of each child was obtained from his mother. Data was collected by means of dietary interview consisting of a 24 hour recall that repeated for 3 days, and a food frequency questionnaire.

Analysis of food items was done using World Food Dietary Assessment System, (WFDAS), 1995, USA, University of California

#### Statistical analysis

All the data were tested for their normal distribution (Kolmogorov–Smirnov test). Results are expressed as means and standard deviation (SD). One way ANOVA was used for comparing variables between different groups using SPSS windows software version 17.0 (SPSS Inc. Chicago, IL, USA, 2008). P values <0.05 were considered statistically significant.

## Results

Table 1 showed the classification of families according to distribution of Obesity among mothers and offspring. Data showed that 8.43% of the families (mother and offspring) were of normal weight, while both of them were obese in 20.48% of studied families. On the other hand in 60.24% of families, mothers were obese, while their offspring were normal. In10.84% of the families, the mothers were obese while offspring were either obese or of normal weight. Data revealed that obesity was high among mothers reached 91.6% while the percent of obesity in the offspring was only 24.5%.

**Table 1:** Classification of Families according to distribution of obesity among mothers and offspring

Family Group	Mothers (Families)		Offspring		Total	

	No.	%	No.	%	No.	%
Group1 Mothers and all offspring are normal weight	7	8.43	17	10.97	24	10.08

Group2 Mothers and all offspring are obese	17	20.48	23	14.84	40	16.81

Group3 Obese Mothers and all offspring are normal weight	50	60.24	88	56.77	138	57.98

Group4 Obese Mothers and their offspring are mixed (normal weight and obese)	9	10.84	27	17.41	36	15.13

			O: 15 N: 12	O: 55.55 N: 44.44		

Total	83	100	155	100	238	100

O: Obese; N: Normal.

Tables (2, 3, and 4) showed the means±SD of the different anthropometric measurements of the mothers, children and adolescents. All the indices of the mothers and children in group (2) were significantly higher compared to other groups, while the mean height of the children in this group was significantly lower (Tables 2, 3). The means ± SD of the adolescent age was varied; group (4) was the older. However the BMI reflect adiposity that showed significant higher values among group (2) and group (4), (Table 4).

**Table 2:** Mean ± SD of Age, Weight, Height and BMI, of mothers according to the different groups

Groups	Group1	Group2	Group3	Group4
Age (yrs)	34.70±6.21	36.40±7.28	32.60±4.51	33.80±4.67

Weight (Kg)	60.20±8.97	99.51±9.52^a^	76.53±9.74^b^	98.10±9.68^c^

Height(Cm)	151.10±11.24	156.02±10.25	154.12±11.37	164.02±10.97^c^

BMI (Kg/m^2^)	24.70±4.56	43.61±5.39^a^	32.31±5.37^b^	36.40±4.78^c^

a Group (1) vs. Group (2);

b Group (1) vs. Group (3);

c Group (1) vs. Group (3) P < 0.00.

**Table 3:** Mean ± SD of age, body weight, height and other anthropometrics indices of the children (<11 years) according to the different groups, (No: 82)

Groups	Group1	Group2	Group3	Group4
Age (yrs)	10.80± 1.01	9.35±2.11	10.43±2.56	10.21±1.20

Weight (Kg)	30.40±2.85	41.80±2.23^a^	34.02±2.85 b	27.51±2.25 c

Height(Cm)	135.04±7.10	123.01±4.12^a^	142.05±8.25 b	130.01±6.23 c

WHZ	-0.21±0.10	-0.72±0.46^a^	-0.44±0.96 b	-0.05±0.09 c

Wt/Ht	0.97±0.22	1.72±0.23^a^	0.93±0.24 b	0.99±0.21 c

BMI (Kg/m^2^)	16.68±3.51	27.63±5.51^a^	16.86±2.39 b	16.27±2.41 c

a Group (1) vs. Group (2);

b Group (2) vs. Group (3);

c Group (2) vs. Group (4) p < 0.00.

**Table 4:** Mean ± SD of Age, Weight, Height and BMI, of adolescent (>11-18) according to the different groups, (No: 73)

Groups	Group1	Group2	Group3	Group4
Age (yrs)	14.01±2.14	16.12±3.17	15.70±2.45	17.01±2.15

Weight (Kg)	48.03±8.27	87.10±7.48^a^	59.40±6.54^b^	83.30±6.71^b^

Height (Cm)	149.05±11.02	159.45±10.12	158.03±12.14	162.20±11.05^b^

BMI (Kg/m^2^)	21.62±4.57	34.41±4.27^a^	23.79±3.71^c^	31.74±4.61^b^

a Group (1) vs. Group (2);

b Group (1) vs. Group (4);

c Group (2) vs. Group (3).

Table 5 showed percent of the frequency consumption of different food items in the all families. Data showed that group (2) revealed high rate intake of all types of food items. Group (1) showed the lower consumption rate of the bread, bakery products, milk, dairy products, eggs, chicken, meat and fish, while showed variable consumption rate in the other items. All groups reported high rate intake of sweets, pastries and beverage.

**Table 5:** The percent of frequency of intakeof different food items in the all families

Food items	Group1		Group2		Group3		Group4	
Intakes /daily	*<3 times %*	*≥3 times %*	*<3 times %*	*≥3 times %*	*<3 times %*	*≥3 times %*	*<3 times %*	*≥3 times %*

Bread & bakery products	69.58	30.42	29.57	70.43	53.26	46.74	32.42	67.58

Milk & dairy products	29.75	70.25	28.36	71.64	24.98	75.02	59.37	40.63

*Intake /weekly*	*<3 times %*	*≥3times %*	*<3 times %*	*≥3times %*	*<3 times %*	*≥3times %*	*<3 times %*	*≥3times %*

Eggs	77.61	22.39	35.47	64.53	46.32	53.68	30.69	69.31

Chicken, meat & fish	68.46	31.54	25.37	74.63	29.65	70.35	26.28	73.72

Legume	47.28	52.72	32.21	67.79	37.63	62.37	62.31	37.69

Vegetables	42.39	57.61	32.24	67.76	27.51	72.49	45.36	95.64

Fruits	36.27	63.73	27.40	72.60	23.58	76.42	40.26	59.74

Sweet, pastries	32.58	67.42	21.30	78.70	36.57	63.43	20.41	79.59

Beverages	31.57	68.43	21.57	78.43	27.91	72.09	19.87	80.13

Tables 6, 7, and 8 showed the mean ± SD of the nutrients intake per day of the mothers and offspring in the different groups. All the mothers consumed diet supply high calories and protein that range from 124.12% to 127.38% and from 182.12% to 196.10% of the RDA, according to the different groups. Group (1) showed the lower consumption rate while group (2) showed the higher one. The same figure was observed as regard the daily intake of carbohydrate, fat and cholesterol. Significant difference was found between the two groups in the intake of protein. Micronutrients in all groups were lower compared to the RDA except sodium and potassium intake.

**Table 6:** Mean ± SD & %of the RDA of the nutrients intake among mothers

Nutrient intake	Group1	Group2	Group3	Group4

	Mean ± S D %RDA	Mean ± S D %RDA	Mean ± S D %RDA	Mean ± S D %RDA
Energy (Cal)	2730.41±248.66	2802.61±98.25	2742.12±68.51	2756.08±323.01
	124.12%	127.38%	124.64%	125.76%

Protein (g)	95.48±27.38	98.05±16.23^a^	95.40±14.20	94.82±27.31
	190.96%	196.10%	190.28%	182.46%

Fat (g)	126.72±29.66	127.43±30.25	124.92±20.33	123.47±29.94

Carbohydrate (g)	297.59±76.63	311.06±64.21	304.98±62.11	311.40±73.37

Dietary fiber (g)	31.03±9.03	30.48±7.29	30.09±8.01	20.75±8.15

Vit. A (μg)	644.87±68.32	567.24±75.34^a^	627.78±187.37	632.67±51.39
	80.61%	70.91%	78.47%	79.08%

Vit. D (μg)	2.03±1.20	1.97±1.36	1.68±0.73	2.79±1.446
	40.60%	39.40%	33.60%	55.80%

Sodium (mg)	636.35±234.82	700.72±47.52^a^	678.25±126.63	594.68±25.14^b^
	127.27%	140.14%	135.65%	118.94%

Potassium (mg)	2180.16±290.58	2229.93±159.37	2350.50±354.21	2216.85±48.06
	109.01%	111.49%	117.53%	110.84%

Calcium (mg)	764.84±434.80	731.63±356.27	617.04±144.74	716.36±37.08
	76.48%	73.16%	61.70%	71.64%

Iron (mg)	9.29±3.57	6.35±2.38	7.95±2.02	8.17±2.11^b^
	61.93%	42.33%	53.00%	81.70%

Zinc (mg)	9.07±3.25	6.27±2.69^a^	6.03±1.57	8.59±4.59^b^
	75.58%	52.25%	50.25%	71.58%

Sat. FA (g)	41.70±13.81	42.91±15.67	42.87±21.30	41.39±11.47

M.uns.f.acids (g)	38.80±20.08	40.09±16.21	36.10±14.27	38.47±11.75

PUFA (g)	35.89±15.47	34.95±11.73	33.97±89.24	34.45±9.92

Cholesterol (mg)	425.64±48.11	437.93±56.24	428.33±105.82	404.47±72.64

a Group (1) vs. Group (2);

b Group (2) vs. Group (4) p < 0.00.

**Table 7:** Mean ± SD & % of the RDA of the nutrients intake among children (up to 11years)

Nutrient intake	Group1	Group2	Group3	Group4

	Mean ± SD %RDA	Mean ± SD %RDA	Mean ± SD %RDA	Mean ± SD %RDA
Energy (Cal)	992.36±31.74^a^	1274.25±29.11	1160.43±36.51^b^	1220.51±34.57^c^
	66.16%	84.95%	77.36%	81.37%

Protein (g)	57.47±12.07	52.63±11.12	56.60±10.20	51.49±8.27
	115.10%	104.97%	112.91%	103.01%

Fat (g)	49.31±6.30	55.17±10.01	52.51±10.24	54.23±7.03

Carbohydrate (g)	79.67±21.30	142.26±30.21^a^	115.36±21.34	131.62±12.17^b^

Dietary fiber (g)	19.55±14.08	14.90±11.27	16.98±12.34	15.01±11.07

Vit. A (μg)	673.97±21.78	570.94±20.31	587.95±37.29	598.94±13.04
	96.28%	81.56%	83.99%	85.56%

Vit. D (μg)	5.33±0.20	4.56±0.52	4.21±0.31	4.23±0.30
	53.30%	45.60%	42.10%	42.30%

Sodium (mg)	145.89±11.02	184.90±20.21^a^	148.91±23.17b^c^	179.01±27.01^b^
	72.94%	92.45%	74.46%	89.51%

Potassium (mg)	670.01±30.27	639.01±20.15	659.03±15.04	634.01±18.01
	95.71%	91.28%	94.14%	90.57%

Calcium (mg)	619.20±30.10	605.01±22.6	619.62±21.07	692.20±16.03^b^
	77.40%	75.62%	77.45%	86.52%

Iron (mg)	8.13±1.34	6.99±1.62	7.03±1.23	7.51±1.07
	81.30%	69.90%	70.30%	75.10%

Zinc (mg)	7.20±1.80	6.75±1.49	6.79±1.09	6.97±1.20
	72.00%	67.50%	67.90%	69.70%

Sat. FA (g)	10.69±2.14	12.30±2.19	11.59±1.03	11.63±2.17

M.uns.f.acids (g)	10.06±1.28	11.50±2.11	11.02±2.09	11.75±1.97

PUFA (g)	7.40±1.97	5.76±2.18^a^	6.46±1.27	6.33±1.62

Cholesterol (mg)	136.40±20.17	184.02±12.54^a^	146.03±14.0^c^	178.39±14.21^b^

a Group (1) vs. Group (2);

b Group (1) vs. Group (4);

c Group (2) vs. Group (3) p < 0.01.

**Table 8:** Mean ± SD & % of RDA of the nutrient intake among adolescent (>11-18 years)

Nutrient intake	Group1	Group2	Group3	Group4

	Mean ± S D %RDA	Mean ± S D %RDA	Mean ± S D %RDA	Mean ± S D %RDA
Energy (Cal)	1058.81±335.99	2718.01±133.10^a^	1260.45±123.28	1596.16±115.66^c^
	52.94%	123.53%	57.31%	79.81%

Protein (g)	43.65±15.11	90.93±372.12^a^	46.89±12.04	49.68±18.17
	87.30%	181.96%	93.78%	99.36%

Fat (g)	43.99±21.44	124.09±31.24^a^	60.98±23.78^b^	34.81±7.47

Carbohydrate (g)	119.46±36.96	309.99±30.77^a^	131.02±29.17^b^	271.04±36.51^c^

Dietary fiber (g)	13.89±7.15	19.01±6.28^a^	15.05±5.27	15.69±8.15

Vit. A (μg)	537.12±321.96	598.12±20.97	589.47±18.74	667.29±214.72^c^
	67.14%	74.77%	73.68%	95.32%

Vit. D (μg)	2.22±0.77	2.16±0.65	3.14±0.14^b^	4.20±14^c^
	44.40%	43.20%	62.80%	84.00%

Sodium (mg)	459.53±211.68	545.25±50.30^a^	432.17±21.34	457.78±116.22
	91.91%	109.05%	86.44%	91.56%

Potassium (mg)	2001.67±190.33	2231.78±45.50	1678.54±33.57	1728.90±34.21
	100.08%	111.59%	83.93%	86.45%

Calcium (mg)	620.41±76.76	424.37±70.61	465.23±25.13	449.14±127.18
	62.04%	42.44%	46.52%	44.91%

Iron (mg)	7.35±3.26	6.13±2.55	6.65±2.47	6.34 ±3.12
	73.50%	61.30%	66.50%	63.40%

Zinc (mg)	7.85±1.60	6.49±2.08	6.97±2.41	6.71±4.29
	65.42%	54.08%	58.08%	55.92%

Sat. FA (g)	20.17±7.87	41.6023±13.59^a^	22.18±6.47	10.03±2.11^c^

M.uns.f.acids (g)	11.60±8.35	37.36±11.75^a^	13.89±6.05	11.97±1.37

PUFA (g)	11.88	34.89±11.69^a^	16.97±6.14	12.84±3.17

Cholesterol (mg)	191.27±122.87	390.58±44.06^a^	210.14±36.25^b^	290.93±56.12^c^

a Group (1) vs. Group (2);

b Group (1) vs. Group (3);

c Group (1) vs. Group (4) p < 0.00.

Children in group (2) showed higher daily intake of calories, and significant higher daily intake of cholesterol and sodium, while all groups showed low levels of daily intake of the micronutrients when compared to the RDA. Adolescents in group (2) showed higher daily intake calories, protein, carbohydrate, fat, cholesterol, sodium, and potassium, while the daily intake of the other micronutrients were low compared to the RDA.

**Figure 1:**
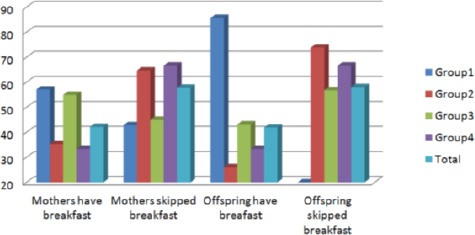
*Percent distribution of mothers and children having or skipped breakfast accordind to different groups*.

Fig. 1 showed the percent of mothers and offspring that used to have or skipped breakfast. Forty-two percent of all the mothers used to consume breakfast, the higher percent (57.14%) were observed in group (1), while groups (2&4) showed the lower percent (35.29% and 33.33%). About 57.83% of all mothers’ skipped breakfast, groups (2&4) showed the higher percent 64.71% - 66.67% respectively. About forty two percent of the all offspring used to consume breakfast; 85.71% was recorded in group (1), while group (2) showed the lower percent (26%). The total percent of offspring skipped breakfast was 58.06%, group (1) showed the lower percent (14.29%) compared to 73.91%, 56.82%, 66.66% in groups (2, 3, 4) respectively.

**Figure 2:**
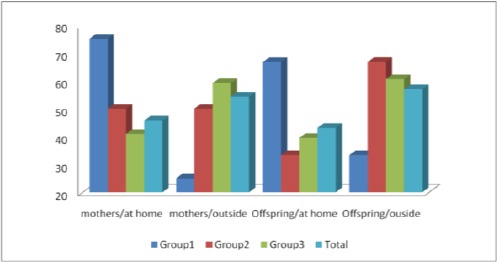
*Place where mothers and offspring get used to take their breakfast, according to different groups*.

Fig. 2 showed the place where mothers and offspring get used to consume their breakfast. Data revealed that the higher percent of mothers and offspring (75%, 66.66%) in group (1) were consumed breakfast inside house compared to the other groups.

## Discussion

Data of this study showed high prevalence of obesity among mother compared to their offspring, 91.57% and 24.5% respectively. Concerning the prevalence of obesity inside the families, data revealed that 20.48% of these families (mothers and offspring) were obese, while 8.43% of them, all were of normal weight. It is interesting here to mention that all mothers in group (3) were obese while all of their offspring (children and adolescents) were of normal weight. In addition, group (4) also revealed that obese mothers with offspring were either obese or of normal weight. The mean values of BMI of the mothers in the three obese groups ranged from 31.31 ± 5.37 to 43.61 ± 5.39. All the mean values of the obesity indices of the children in group (2) including the WHZ, Wt/Ht and the BMI were significantly high compared to the other groups, while the mean BMI values in the adolescents were significantly higher in groups (2&4).These results may lead us to suggest that obesity was more prevalent among adolescents than children.

The food pattern of the families showed variations in the rate of consumption of the different food. The high rate of sweet, pastries and beverages consumption that were reported in all groups could be the reason for the high daily caloric intake observed in the diet of all mothers and adolescent especially in group (2). Consumption of different food supplied from either plant or animal protein like legumes, eggs, meat, chicken and fish were balanced and complemented each other, as was expressed in the high protein content in their daily intakes. In this context the significant differences between group (1) and group (2) highlight the importance of the food pattern consumption as the cause of obesity, especially their daily intake of calories and protein. At the same time the hereditary factors must be overloaded. However 23.58-45.36% of the families in the different groups consumed vegetables and fruits at lower rate, as were the rate of milk and milk products consumption which ranged from 24.98-59.37% and 40.638-75.02% respectively, whicheffecting negativelytheir levels of daily intake of vitamins and minerals.

Adolescents acquired an overall low quality food consumption pattern as was depicted in this study especially in group (4) where obesity was present among adolescents compared to children. Eating occasions for many early adolescents (11–14 years) are characterized by poor overall diet quality and overconsumption of energy [15, 16]. Fruit and vegetable [17], and whole grain and fiber consumption is low [18, 19], and intake of sodium and calories from added sugars is high [20-23]. Eating occasions in line with the information obtained from this study in group (4) where obesity was more among adolescents compared with children.

Mother’s daily intake of vitamin D was low in all groups compared to RDA, it ranged from 33.60% to 55.8o%. In the same time their daily intake of calcium also was low in the all groups which ranged from 61.70% to 76.48% that reflected their low daily milk and dairy products intake. The same pattern was observed in their offspring. Liu and his colleagues, 2016 [24], reported that children and adolescents suffering from obesity are more likely to have lower concentrations of serum 25(OH) vitamin D.

Moreover, the prevalence of vitamin D deficiency was associated with obesity in Asians and European-American, [25]. In addition, Yusr et al.(2014) [26] stated that a lower vitamin D serum level could be a modifiable risk factor for obesity, insulin resistance and cognitive impairment in middle age Egyptian females. An area of particular interest in current obesity research is the potential association between calcium intake and body weight. Inverse associations have been reported between calcium intake and body weight in both retrospective, [27-30], and prospective analyses [31]. Some studies have shown that calcium is inversely associated with body fat percentage, [29, 31, 32]. Additional studies have found an inverse relationship between calcium intake and of being overweight or obese [28, 33], as well as between calcium intake and abdominal adipose tissue [29]. However two recent randomized control trials revealed no significant trends in the direction of a positive effect of calcium intake on weight loss [34, 35]. In this context our data were in line with the last information and did not proof such a positive relation in the non-obese individuals, taking in consideration the results of dietary examination.

One of our interests in this study was to investigate the effect of breakfast on the prevalence of obesity in families, as many studies reported this relationship. Szajewska and Ruszczynski (2010) [36] showed that the effect of eating breakfast on the body mass index (BMI) in children and adolescents in Europe was analyzed in 4 studies (n = 2897). All of these studies showed an increase in BMI in breakfast skippers. de la Hunty et al (2013), [37] reported that, the evidence reviewed is suggestive that regular consumption of breakfast cereals results in a lower BMI and a reduced likelihood of being overweight in children and adolescents. However, more evidence from long-term trials and investigations into mechanisms is needed to eliminate possible confounding factors and determine causality. Results of this study were in line with the previous study, data showed that 57.83% of mothers and 58.o6% of offspring skipped breakfast. The lower number of mothers skipped breakfast was found in the first group (42.86%), while the higher was reported in group (2 & 4) as it reached 64.71% and 66.67%. For offspring 14.29% of the offspring in group (1) skipped breakfast compared to 73.91% in group (2). Group (3 & 4) also showed higher percent 56.82% and 66.66% respectively. In addition data revealed that the higher percent of mothers and offspring who used to take breakfast inside house was (75%, 66.66%) in group (1) compared to the lower percent in other groups. These results proved the effect of dietary habits between mothers and offspring, mothers and children who eat their breakfast inside home had normal weights, which add more support concerning eating inside the home is more balanced and healthy

In conclusion, the results of this study suggested that family environment is an important influence on the dietarybehaviorsof young people mainly adolescentsas a considerable sector of families. Unhealthy dietary pattern especially caloric dense food and skipping breakfast, or used to take breakfast outside house is associated with risk of becoming overweight or obese.
